# Complete Genome Sequence of Stenotrophomonas maltophilia Siphophage Suso

**DOI:** 10.1128/mra.00117-22

**Published:** 2022-03-14

**Authors:** Qori Emilia, Kyra Groover, James Clark, Tram Le, Ben Burrowes, Mei Liu

**Affiliations:** a Department of Biology, Texas A&M University, College Station, Texas, USA; b Center for Phage Technology, Texas A&M University, College Station, Texas, USA; c Department of Biochemistry and Biophysics, Texas A&M University, College Station, Texas, USA; d BB Phage Consultancy, LLC, Georgetown, Texas, USA; Portland State University

## Abstract

Phage Suso is a temperate siphophage of Stenotrophomonas maltophilia with a 44,659-bp genome. This phage is closely related to *Stenotrophomonas* phage SM171, sharing 92% overall nucleotide identity as determined by BLASTn, and it shares 14 similar proteins (BLASTp, E value < 0.001) with some *Pseudomonas* phages from the genus *Beetrevirus*.

## ANNOUNCEMENT

Stenotrophomonas maltophilia is emerging as a multidrug-resistant respiratory pathogen (1) and is associated with lethal bacteremia in immunocompromised people (2). It is necessary to develop alternative treatment for S. maltophilia infections, including using phage as therapeutics. Phage Suso was isolated against S. maltophilia, and its genome features are reported here.

Phage Suso was isolated from a freshwater sample collected from the Colorado River region (approximate GPS coordinates, 28.789140, −95.997233), in Wadsworth, TX, in September 2019, using S. maltophilia strain ATCC 17807 as a propagation host. Phage were purified by three rounds of picking clear plaques and propagating using a soft agar overlay method (3) with the host strain grown aerobically in tryptone nutrient broth/agar at 30°C. Phage morphology was determined by imaging negatively stained samples using 2% (wt/vol) uranyl acetate (4) through transmission electron microscopy (TEM) at the Texas A&M Microscopy and Imaging Center. Phage genomic DNA was purified by precipitating phage particles using polyethylene glycol (PEG) followed by a Promega Wizard DNA cleanup system as described previously (5). DNA sequencing libraries were prepared using a Swift 2S Turbo kit as 300-bp inserts and sequenced on an Illumina MiSeq machine with paired-end 150-bp reads using V2 300-cycle chemistry. A total of 183,832 raw reads were obtained and were quality controlled with FastQC (www.bioinformatics.babraham.ac.uk/projects/fastqc) and trimmed with FASTX-Toolkit v0.0.14 (http://hannonlab.cshl.edu/fastx_toolkit/). The trimmed reads (87,654 reads) were used for assembly using SPAdes v3.5.0 (6). A contig at 139-fold coverage was obtained, and its end sequences were amplified by PCR and confirmed to be complete by Sanger sequencing of the PCR product using primers (forward, 5′-GATCGGATTGCCGTTGTTCG-3′, and reverse, 5′-GTCGACATGGAGCTGTGGG-3′). Annotation of the phage genome was performed using the Center for Phage Technology (CPT) Galaxy-Apollo platform (https://cpt.tamu.edu/galaxy-pub) (7–9). Gene calling was performed using Glimmer v3.0 (10) and MetaGeneAnnotator v1.0 (11). tRNA genes were detected with ARAGORN v2.36 (12) and tRNAScan-SE v2.0 (13). Gene functions were predicted using InterProScan v5.48 (14), BLAST v2.9.0 (15), TMHMM v2.0 (16), HHPred (17), LipoP v1.0 (18), and SignalP v5.0 (19). BLAST was carried out against the NCBI nonredundant (nr) and SwissProt databases (20). Phage termini were predicted using PhageTerm (21). Genome-wide DNA sequence similarity was calculated by ProgressiveMauve v2.4 (22). All software was run at default settings.

**FIG 1:**
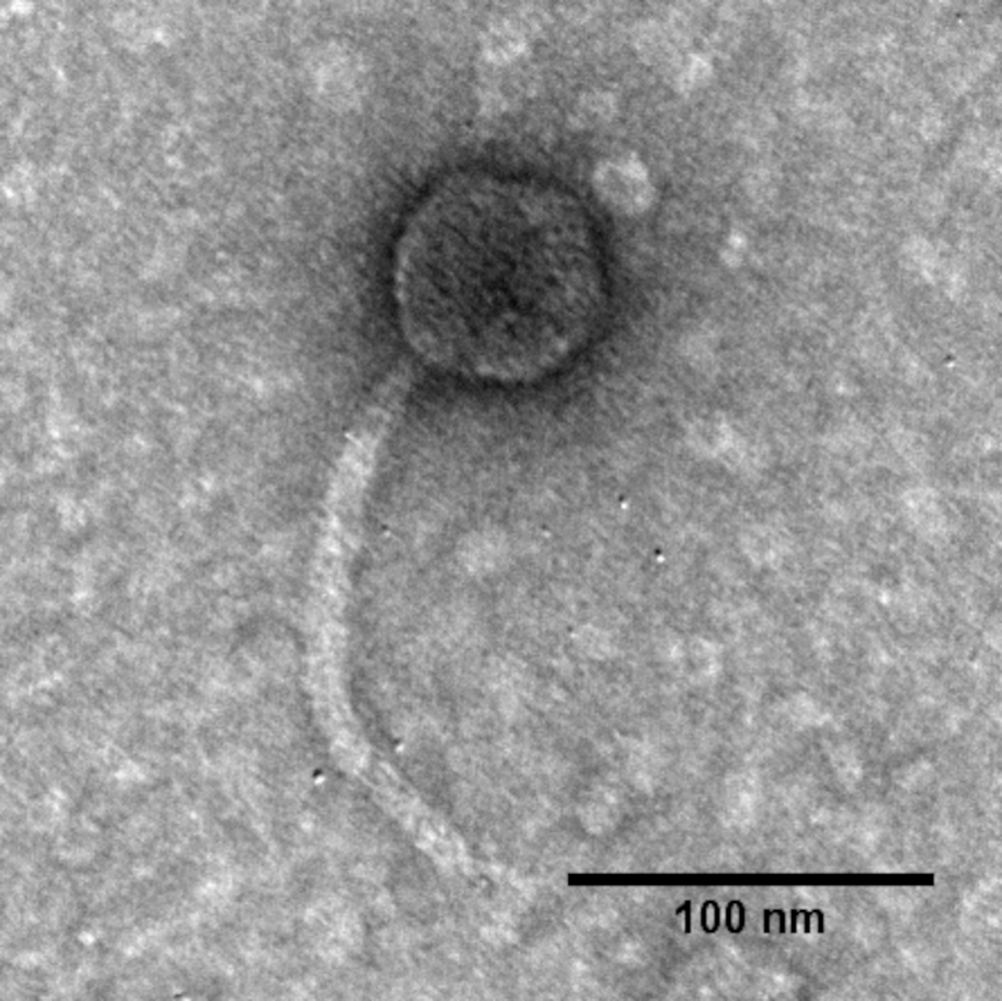
Transmission electron micrograph (TEM) of phage Suso. Phage particles were diluted with TEM buffer (20 mM NaCl, 10 mM Tris-HCl [pH 7.5], 2 mM MgSO_4_), captured on carbon films, negatively stained using 2% (wt/vol) uranyl acetate, and observed on a JEOL 1200 EX TEM at a 100-kV accelerating voltage at the Microscopy and Imaging Center at Texas A&M University.

Phage Suso has a siphophage morphology (Fig. 1) possessing a 44,659-bp genome with a GC content of 67%. This phage has 69 protein-coding genes, of which 26 have predicted functions, and no tRNA genes. A headful or pac packaging mechanism was predicted using PhageTerm (21). Genes related to lysis, replication, recombination, regulation, and structure were identified. A lysis cassette was identified encoding an endolysin with endopeptidase activity, two potential holin/antiholins, one with class IV and one with class I topology, an i-spanin, and an o-spanin. The presence of a gene encoding an immunity repressor indicates a potential temperate lifestyle for this phage. At the time of this submission, phage Suso is closely related to only one phage in the NCBI database, *Stenotrophomonas* phage SM171 (MZ611865), sharing 92% overall nucleotide identity as determined by BLASTn. Besides this phage, Suso shares 14 similar proteins (BLASTp, E value < 0.001) with some *Pseudomonas* phages from the genus *Beetrevirus* (taxid 2560098), such as phages B3 (GenBank accession number NC_006548), JBD67 (GenBank accession number NC_042135), and vB_Pae_BR141c (GenBank accession number MK511065).

### Data availability.

The Suso sequence was deposited in GenBank with accession number MZ326866. The associated BioProject, SRA, and BioSample accession numbers are PRJNA222858, SRR14095259, and SAMN18509666, respectively.
